# Changes in Oral Mucosa Associated with Melanotan II Injections: A Case Report

**DOI:** 10.3390/life16020265

**Published:** 2026-02-03

**Authors:** Alexander Bonchev

**Affiliations:** Department of Conservative Dentistry, Faculty of Dental Medicine, Medical University, 1431 Sofia, Bulgaria; a.bonchev@fdm.mu-sofia.bg

**Keywords:** Melanotan II, oral pigmentation, gingival discoloration, unregulated peptides, case report

## Abstract

This case report presents a three-month follow-up of a patient who self-administered Melanotan II injections over a period of 64 days with the goal of achieving a deeper tanning effect. Melanotan II is an unlicensed synthetic peptide analog belonging to the melanocortin hormone family. It acts primarily by activating melanocortin 1 receptors on melanocytes, stimulating eumelanin production and resulting in skin pigmentation independent of sun exposure. Despite its popularity, particularly through promotion on social media, Melanotan II remains unregulated, and its use is associated with a range of potential adverse effects. During the initial intraoral examination, brown pigmentation was observed on the attached gingiva in both the maxillary and mandibular arches. The lesions were almost symmetrically distributed, with a more intense coloration in the anterior region of the lower jaw. Additional pigmented areas with irregular shapes and poorly defined borders were noted on the left and right buccal mucosa. At the one-month follow-up after discontinuation of the injections, the buccal mucosal pigmentation had nearly disappeared. However, at the three-month follow-up, gingival pigmentation persisted, though with visibly reduced intensity. To date, there is a lack of published data specifically addressing the timeline for resolution of oral pigmentation associated with Melanotan II use, making this case a valuable contribution to the limited existing literature on the subject.

## 1. Introduction

Melanotan II is an unlicensed synthetic peptide analog that falls within the melanocortin family of hormones. It is a cyclic lactam derivative (Ac-Nle-α-MSH-NH2) designed to mimic the effects of α-melanocyte-stimulating hormone (α-MSH). By activating the MC1-R receptors on melanocytes, it promotes the production of eumelanin, leading to a tanned appearance without exposure to sunlight [1,2,3]. Melanotan II is accessible in both injectable and nasal spray forms. Although it remains unregulated and illegal in Bulgaria, as well as in numerous other countries, its use has increased significantly in recent years. This rise in popularity is largely fueled by social media content showcasing its ability to produce quicker and more intense tanning effects [4]. Several studies in the literature have explored the potential association between Melanotan use and the development of cutaneous melanoma [3,5,6]. However, conclusive evidence confirming a direct causal relationship remains lacking. In addition to its effects on the skin, Melanotan II has also been associated with intraoral pigmentation. A recent report described a case of intraoral melanoma potentially linked to the use of a nasal spray containing Melanotan II [7].

The primary objective of the present clinical case is to monitor and document intraoral mucosal changes before, during, and up to three months after the administration of Melanotan II. To the best of the authors’ knowledge, this is the first documented follow-up case assessing oral mucosal reactions to this peptide. An additional aim of this report is to raise awareness among dental professionals, who may be the first to observe such mucosal changes and are therefore in a unique position to inform patients about the potential risks associated with Melanotan II use.

## 2. Case Presentation

A 42-year-old Caucasian male presented to the dental office for a routine dental examination. He reported no known systemic health conditions and was not taking any medications regularly. Family history revealed that both his parents and grandparents were also of Caucasian origin. The patient reported a history of tobacco use. The patient reported daily gym training over an extended period and adherence to a low-carbohydrate, high-protein, and high-fiber diet. He also admitted to regular use of sunbeds.

**Figure 1:**
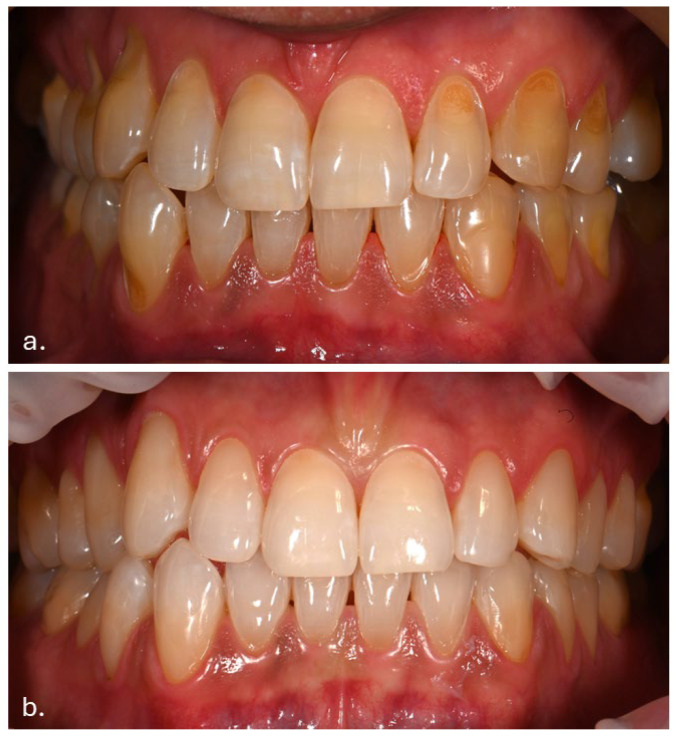
(**a**) Intraoral photograph taken during the patient’s initial examination, showing cervical abfractions. (**b**) Post-treatment photograph following restoration of the abfractions with direct composite resin.

The patient had previously received treatment at the same practice and was familiar to the dental team. Several cervical abfractions had been restored with composite resin restorations. During these earlier visits two years ago, a slight brown pigmentation was noted on the attached gingiva in the lower anterior region. In contrast, the gingiva of the maxillary arch and the oral mucosa in other regions appeared clinically normal, with no evidence of pigmentation (Figure 1).

However, during the current appointment, his skin exhibited a noticeably darker tan than previously observed. When questioned about the change, the patient disclosed that he had been administering Melanotan II over the past month to enhance tanning and improve visual muscle definition. The patient reported using Melanotan II peptide (10 mg/2 mL) purchased from an online retailer specializing in research peptides. He self-administered the peptide subcutaneously in the lower abdomen at a dose of 400 micrograms every other day over a 64-day period, for a total of 32 injections and a cumulative dose of 12.8 mg. Concurrently, he underwent 5 sunbed sessions, each lasting 24 min, in an effort to enhance the tanning effects.

**Figure 2:**
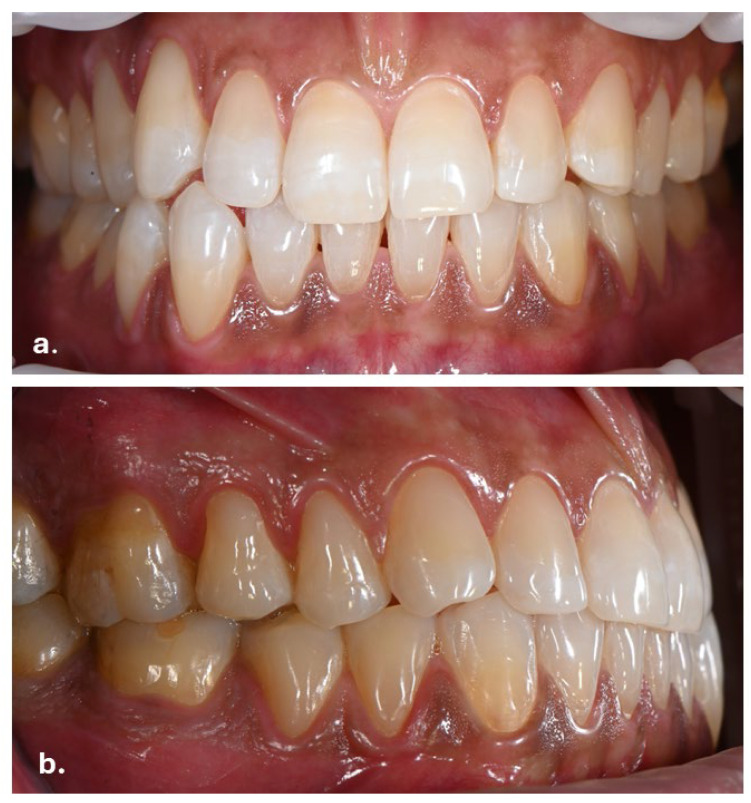
Symmetrical brown pigmentation of the attached gingiva during the Melanotan II therapy: (**a**). frontal view; (**b**). lateral view.

Intraoral examination revealed brown pigmentation of the attached gingiva in both the maxillary and mandibular arches. The lesions were almost symmetrically distributed and more intensive in color in the anterior region of the lower jaw. Areas with previously observed pigmentation appeared darker in color and showed an increase in surface area. The pigmentation did not extend to the tips of the interdental papillae (Figure 2).

**Figure 3:**
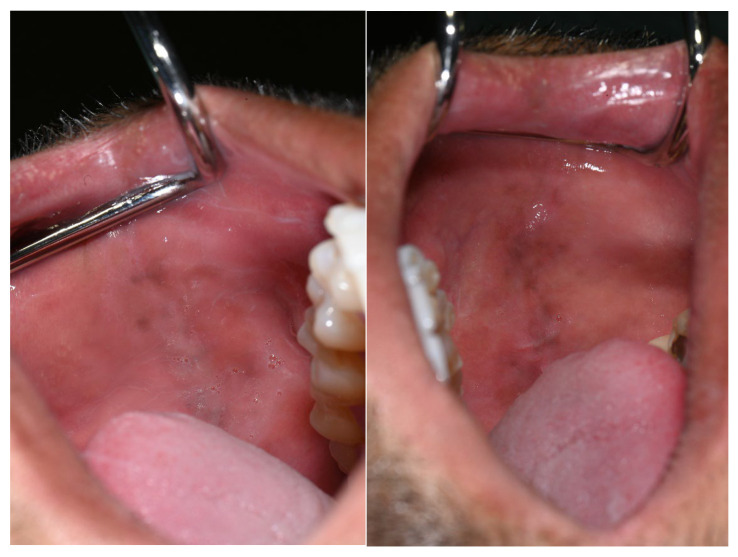
Pigmentations on the buccal mucosa during the application of Melanotan II.

Additional pigmented areas were observed on the left and right buccal mucosa with irregular shape and poorly defined borders (Figure 3).

**Figure 4:**
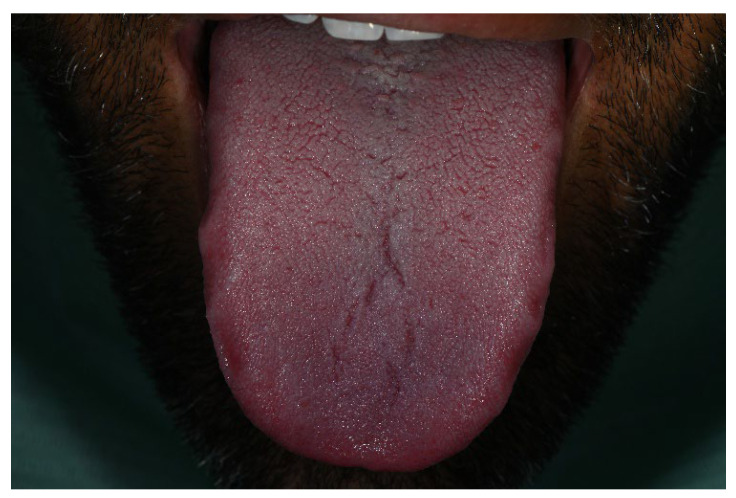
No pigmentation was observed on the tongue during the period of Melanotan II administration.

Notably, the tongue, palatal mucosa, and lips showed no signs of pigmentation (Figure 4).

The patient reported an increase in libido during the period of Melanotan II use but did not experience other common adverse effects such as nausea or reduced appetite. The patient also noted darkening of some pre-existing melanocytic skin nevi, without any associated changes in size, shape, or surface characteristics. During the consultation, the patient was thoroughly informed about the potential risks and side effects associated with Melanotan II use. He mentioned his intention to undergo and finish another course using a pre-filled syringe. Following the discussion, he was advised to discontinue further injections and avoid solariums. Following the dental consultation, the patient discontinued Melanotan II immediately.

Follow-up visits were scheduled at 14-day intervals to enable close clinical surveillance in the absence of established guidelines for Melanotan II-associated oral pigmentation and to maintain a low threshold for biopsy should concerning changes arise. At each visit, lesion size, color intensity, distribution, symmetry, and the presence of features suggestive of malignancy or other significant pathology were systematically assessed. No clinically significant changes were observed during the interim visits. Therefore, the case report emphasizes the follow-up time points at which clinically visible changes occurred, namely 28 days and three months after cessation of Melanotan II. In addition, the patient was instructed to perform regular self-monitoring of the oral lesions and to promptly contact the treating clinician in the event of any negative or concerning changes.

**Figure 5:**
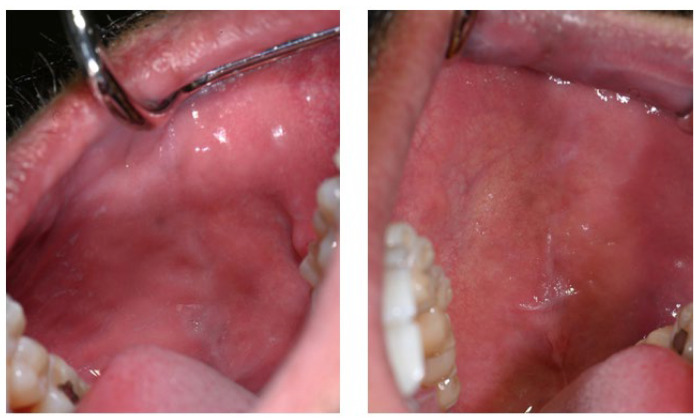
Buccal mucosa 28 days after discontinuation of Melanotan II application.

The first noticeable signs of size reduction and decreased pigmentation intensity were observed after 28 days, most prominently on the buccal mucosa (Figure 5).

**Figure 6:**
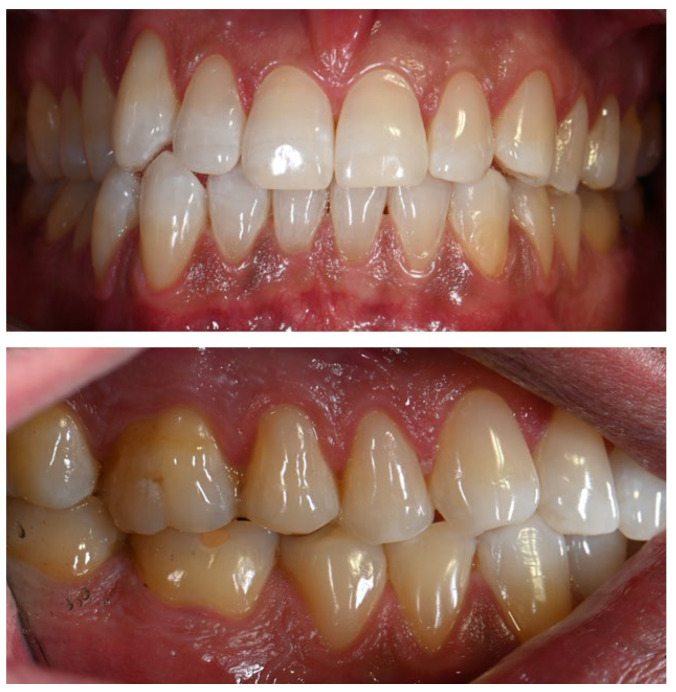
Marked reduction in brown pigmentation, although the mucosal appearance had not completely reverted to its pre-Melanotan II condition.

Three months after cessation of Melanotan II therapy, gingival pigmentation had diminished but was not completely resolved (Figure 6).

**Figure 7:**
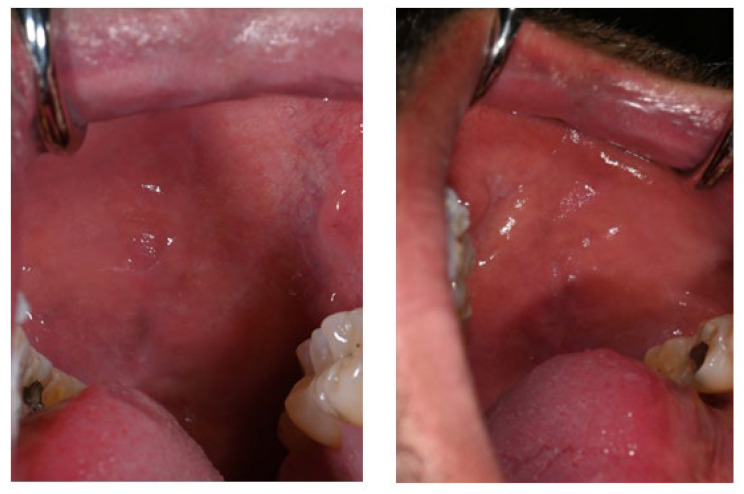
Clinical appearance of the buccal mucosa three months following discontinuation of Melanotan II therapy.

The buccal mucosa demonstrated marked improvement compared with its appearance during active therapy (Figure 7).

To provide a clearer and more objective clinical characterization of the lesions and their evolution over time, a structured summary of the anatomical distribution, clinical appearance, periodontal status, and corresponding photographic documentation is presented in Table 1. This timeline allows correlation of clinical findings with Melanotan II exposure and subsequent follow-up.

## 3. Discussion

At the patient’s initial presentation two years prior, light brown pigmentation was noted on the labial aspect of the attached gingiva in the anterior mandibular region. One possible explanation for this pigmentation was physiological melanosis, which is typically benign and results from increased melanin production by melanocytes [8,9]. The color of such pigmentation can range from light brown to nearly black. However, this diagnosis is less likely in this case given the patient’s Caucasian background, as physiological pigmentation is more commonly observed in individuals with darker skin tones [8]. An alternative etiology for the initial pigmentation is smoker’s melanosis, a condition observed in approximately 25–31% of tobacco users [8]. It presents as discrete or confluent brown macules, often localized to the labial gingiva of the mandible. The patient reported long-term, regular tobacco use, further supporting this possible explanation.

In the current presentation, however, there was a marked progression in both the number and size of the pigmented lesions with a notable increase in pigmentation intensity. It is well-documented that the oral pigmentation can be influenced by physical, chemical, and hormonal factors [10], as appears to be the case here. Given the absence of systemic diseases and the lack of reported use of other medications, an association was observed between the recent changes in oral pigmentation and the use of Melanotan II. However, this finding must be interpreted with caution due to pre-existing gingival pigmentation and potential confounding factors, including smoking and sunbed use.

A variety of conditions can lead to abnormal pigmentation within the oral cavity, including Addison’s disease, Peutz–Jeghers syndrome, melanoacanthoma, foreign body reactions, heavy metal exposure, amalgam tattoo, and pigmented neoplasms [10]. Accurate differentiation among these conditions cannot be based on clinical appearance alone and typically requires correlation with medical history, laboratory investigations, and, when indicated, histopathological examination within a multidisciplinary diagnostic approach.

Drug-induced oral pigmentation has also been reported with certain medications, such as mynocicline, antimalarials, amiodarone, clofazimine, chemotherapeutics, clofazimine, hormones, ketoconazole, oral contraceptives, and zidovudine [11,12]. These agents are known to produce a characteristic grayish-blue discoloration, most commonly affecting the hard palate and gingiva. Notably, such pigmentation is often idiosyncratic and does not show a clear correlation with the dosage or duration of the drug regimen [13]. The microscopic appearance of drug-induced oral pigmentation varies depending on the specific medication. It can resemble a melanotic macule, showing melanin accumulation in the basal cell layer, or it may result from the deposition of drug-related metabolites as fine brown-yellow granules within the lamina propria [11,12]. In the case of minocycline, pigmentation typically appears as a brown to black discoloration of the gingiva in both the upper and lower jaws, often due to staining of the underlying bone rather than the soft tissues [12].

Drug-induced pigmentation of the oral mucosa typically resolves within a few weeks to several months following the discontinuation of the causative agent, although in some cases, the discoloration may persist permanently [10]. A biopsy should be considered when the pigmentation occurs in areas with a higher risk for melanoma, such as the maxillary gingiva or palate, especially if a clear link to the medication cannot be confirmed [12]. Nevertheless, drug-induced pigmentation has not been reported to undergo malignant transformation [14,15].

Data on the duration required for skin pigmentation to normalize after Melanotan II use remain scarce. In a case described by Ong and Bowling, the patient’s skin discoloration began to diminish approximately two weeks after the final injection, with full return to baseline pigmentation observed within one month. Although the patient reportedly received seven injections at weekly intervals, specific details regarding the dosage and concentration of the administered product were not disclosed. In the present case, complete recovery of the normal mucosal coloration was not observed after 3 months. To date, there is a lack of published data specifically addressing the timeline for resolution of oral pigmentation associated with Melanotan II use, making this case a valuable contribution to the limited existing literature on the subject.

In the early 1980s, researchers (Hadley and Dorr) developed a synthetic analog of α-melanocyte-stimulating hormone (α-MSH) known as [Nle4-D-Phe7]-α-MSH. Initially named Melanotan or Melanotan I, this compound is now referred to by its generic name, afamelanotide. It has shown therapeutic promise in treating several dermatological conditions, including vitiligo, polymorphic light eruption, erythropoietic protoporphyria, solar urticaria, and Hailey–Hailey disease [3,16,17,18,19]. A related compound, Melanotan II, is a newer, shorter cyclic version of α-MSH that appears to have enhanced potency. Like afamelanotide, Melanotan II influences skin pigmentation, but it has also been associated with side effects such as nausea and spontaneous penile erections [5,20,21]. Interestingly, its impact on sexual arousal led to clinical studies exploring its potential in treating erectile dysfunction [22].

Melanotan II is a synthetic analog of α-melanocyte-stimulating hormone (α-MSH) and may influence oral mucosal pigmentation through stimulation of the melanocortin 1 receptor (MC1R) on oral melanocytes. Activation of MC1R has been associated with upregulation of melanogenesis, potentially leading to increased melanin synthesis and transfer of melanosomes to adjacent keratinocytes, which could contribute to the development of visible mucosal pigmentation [23,24].

Pigmentation appears to occur more frequently in the attached gingiva than in the marginal gingiva, which may be related to differences in epithelial architecture and melanocyte distribution. The attached gingiva is characterized by a thicker, keratinized epithelium and a relatively higher density of melanocytes, possibly making it more responsive to MC1R stimulation. In contrast, the marginal gingiva is thinner and less keratinized and may therefore be less likely to exhibit pronounced pigmentation [25,26].

The symmetric distribution of pigmentation may be explained by the systemic administration of Melanotan II, resulting in relatively uniform exposure of MC1R-expressing melanocytes across bilateral oral sites. This symmetry likely reflects both the widespread distribution of melanocytes and the systemic nature of the compound’s effects [26].

At the tissue level, oral pigmentation may involve increased melanin production within basal layer melanocytes, followed by transfer of melanosomes to suprabasal keratinocytes [14,23,24,25]. Concurrent ultraviolet exposure is unlikely to play a significant role in oral mucosal pigmentation, as the oral cavity is largely shielded from UV radiation.

Nevertheless, due to the mentioned side effects, such as skin darkening and appetite suppression leading to weight loss, Melanotan II has gained the nickname “the Barbie drug.” Multiple case reports have described serious adverse effects linked to the use of Melanotan II, including the development of exogenous hypercortisolism, rhabdomyolysis, priapism and acute renal failure [2,27,28]. Several regulatory authorities, including the U.S. Food and Drug Administration (FDA), the Health Products Regulatory Authority (HPRA) in Ireland, and the Therapeutic Goods Administration (TGA) in Australia, have issued warnings regarding the potential significant safety risks associated with the use of Melanotan II [29,30,31].

The primary motivation for the patient’s use of Melanotan II was to achieve a deeper tan and enhance visual muscle definition, which aligned with his long-term gym training and fitness-oriented lifestyle. This effect was further intensified by the concurrent use of sunbeds both before and during the injection period. The tanning industry plays a significant role in promoting such practices, particularly through aggressive marketing on social media platforms like Facebook and Twitter, which are popular among adolescents and young adults. These campaigns often feature special offers and encourage frequent usage [32]. Therefore, in recent years, media coverage has increasingly highlighted concerns related to the use of nasal tanning sprays. For instance, an investigation by the BBC involved purchasing 10 such products from both online retailers and physical locations, including beauty salons and tanning shops. Laboratory analysis revealed that six of these sprays contained Melanotan II, with varying concentrations of the substance detected [33].

Distinguishing benign, drug-induced oral pigmentation from malignant or systemic causes is essential, as serious conditions such as oral mucosal melanoma may present with similar clinical features [12,14,26]. Diagnostic evaluation begins with a thorough clinical history, including medication use (notably Melanotan II), timing of onset, lesion evolution, and associated systemic symptoms. In cases where pigmentation is symmetric, non-tumefactive, clinically stable, and temporally associated with drug exposure, conservative management with close clinical monitoring may be justified [12,14,34,35]. This approach is further supported when lesions demonstrate regression following cessation of the suspected agent, as observed in the present case, where buccal pigmentation with poorly defined borders began to fade after discontinuation of Melanotan II.

Current literature recommends regular clinical follow-up for drug-related pigmentation, typically at 12-month intervals, combined with patient self-examination every 3–4 months [34]. Nevertheless, vigilance is required, and biopsy or further diagnostic work-up should be considered if concerning features emerge. Red-flag criteria include rapid lesion growth, marked asymmetry, ulceration or spontaneous bleeding, nodularity, color variegation, induration, fixation to underlying tissues, pain, or associated systemic symptoms, all of which warrant prompt referral and histopathological evaluation to exclude malignancy [12,14,34,35,36,37].

A recent case report by Alsabbagh et al. is believed to be the first documented instance of oral mucosal melanoma linked to the nasal spray form of Melanotan II. The case presented a 22-year-old white female who had been using a Melanotan II nasal spray prior to UV tanning sessions, administering two sprays into each nostril twice daily over the course of one month. Within three weeks of beginning the product application, the patient noticed pigmentation on her gums along with the emergence of a swelling. Histopathological evaluation confirmed a pT4 melanoma infiltrating the alveolar bone, with a depth of invasion measuring 9 mm [7].

A review of the existing literature identified four reported cases of melanoma potentially associated with the use of Melanotan II, all of which involved subcutaneous administration [3,5,6,38]. However, the likelihood of a direct causal relationship between Melanotan II and melanoma development remains uncertain. Animal studies, including in vivo experiments and murine models, have not demonstrated carcinogenic properties for Melanotan I, the linear analog of α-MSH. Notably, α-MSH itself has shown both protective and potentially harmful effects. Some studies suggest it has anticarcinogenic properties and may act as a tumor suppressor. Conversely, other research indicates that α-MSH can promote invasive behavior in melanoma cells, potentially aiding in immune evasion [5,39].

Despite limited scientific evidence, the potential risks associated with the use of experimental substances like Melanotan II must be emphasized, as their safety profiles remain unverified. To date, the literature contains only a single case report describing generalized intrinsic blue-brown discoloration or hyperpigmentation of the gingiva following six months of subcutaneous self-injection with Melanotan, undertaken to achieve a cosmetically desirable tan. However, that report lacked detailed information regarding dosage and frequency of use [16]. In contrast, the present case provides detailed exposure information together with structured photographic documentation obtained before, during, and at defined intervals after cessation of Melanotan II injections. This visual follow-up allows observation of the temporal behavior of oral pigmentation, notably demonstrating almost complete resolution of buccal mucosal pigmentation with persistence of gingival discoloration over time—an aspect not previously characterized in published reports.

Management of oral pigmented lesions should be guided by a thorough and accurate diagnostic process to ensure appropriate treatment planning. Among the available therapeutic options, surgical laser therapy has been reported to be well tolerated by patients [40] and effective in the management of physiological gingival pigmentation, smoker’s melanosis, and pigmentation associated with Laugier–Hunziker syndrome. Different laser modalities, such as CO_2_, Er:YAG, and diode lasers, have demonstrated comparable clinical outcomes in the treatment of oral pigmented lesions [41].

To date, there are no published data specifically addressing the management of persistent oral pigmentation associated with Melanotan II use. Consequently, any therapeutic considerations in this context should be approached with caution, and further well-documented clinical observations and controlled studies would be necessary before drawing conclusions regarding the efficacy or safety of potential treatment modalities.

## 4. Conclusions

Raising awareness of such adverse effects within the dental community is crucial. In this particular case, the patient was unaware of the potential side effects and was considering resuming injections. Thanks to timely counseling by the dentist regarding the possible health risks, the patient decided to discontinue use. This highlights the important role dental professionals can play in recognizing and addressing the consequences of unregulated substance use.

## Figures and Tables

**Table 1:** Clinical timeline and evolution of the oral pigmentation.

Timing Relative to Melanotan II Use	Clinical Findings	Corresponding Figures
Two years before Melanotan II use	Diffuse, uneven brown pigmentation confined to the anterior vestibular attached gingiva of the mandibular arch. No pigmentation of the maxillary gingiva or buccal mucosa. Gingiva clinically healthy, with normal contour and consistency, no erythema or edema, no bleeding on probing, and no periodontal pocketing.	Figure 1
During Melanotan II use	Bilateral, almost symmetric brown macular pigmentation of the attached gingiva in both arches, more intense in the anterior mandibular region, with enlargement and darkening of previously pigmented areas. Pigmentation spared the interdental papillae. Additional irregular, poorly defined pigmented macules were present bilaterally on the buccal mucosa, predominantly at the level of occlusal contact between the maxillary and mandibular teeth. Several macules showed partial confluence. Lesions were flat (non-tumefactive), asymptomatic, and without ulceration or bleeding. Tongue and palate were unaffected. Periodontal tissues remained clinically healthy.	Figure 2, Figure 3 and Figure 4
28 days after cessation	Gingival pigmentation remained present with no immediate reduction in size or intensity at early follow-up. No increase in size or new lesions observed. Initial signs of regression were noted, characterized by a decrease in pigmentation intensity and surface area, most prominently on the buccal mucosa. Gingival tissues remained clinically stable and non-inflamed.	Figure 5
Three months after cessation	Further fading of gingival pigmentation, without complete resolution. Marked improvement of buccal mucosal pigmentation was observed compared with findings during active therapy, with complete resolution of some macules and reduced color intensity of the remaining isolated macules. Periodontal status remained unchanged and clinically healthy.	Figure 6 and Figure 7

## Data Availability

The data presented in this study is available in this article.
